# Serum Vitamin D Levels in Autoimmune and Non-Autoimmune Hypothyroidism: A Retrospective Study from Jordan

**DOI:** 10.1055/s-0045-1811590

**Published:** 2025-10-14

**Authors:** Hadeel Alqurieny, Mohammad Al-Slehat, Karam Bdour, Roa'a Abedel Razaq Abu Lail, Abdel Qader Abu-Salih, Zaid Iyad Mohammad Aldebei, Mohammad Al-Bdour, Rula Al Shimi, Asmaa Quraan, Abdel Razaq Al Yasin, Fadi Ayyash

**Affiliations:** 1 Department of Endocrinology, Queen Rania Al Abdullah Hospital for Children, Royal Jordanian Medical Services, Amman, Jordan; 2 Departmant of Internal Medicine, Jordan University Hospital, Amman, Jordan; 3 Department of Endocrinology, King Hussein Medical Center, Amman, Jordan; 4 Faculty of Medicine, Al-Balqa Applied University, Al-Salt, Jordan; 5 Faculty of Medicine, Jordan University of Science and Technology, Irbid, Jordan

**Keywords:** autoimmune hypothyroidism, non-autoimmune hypothyroidism, 25-hydroxyvitaminD, FT4

## Abstract

**Background:**

Vitamin D is a steroid hormone primarily produced in the skin. In Jordan, vitamin D deficiency is widespread among the population. This study aims to compare serum vitamin D levels between patients with autoimmune and non-autoimmune hypothyroidism.

**Methods:**

A retrospective observational study was conducted at the Jordanian Royal Medical Services in Jordan from January 2023 to November 2024. Data were gathered from the patient's medical records, including age, gender, vitamin D level, thyroid-stimulating hormone (TSH) value, free thyroxine (FT4) level, anti-thyroid peroxidase, and anti-thyroglobulin levels.

**Results:**

A total of 150 patients were included, aged 5 to 76 years, with a mean age of 39.2 years. The mean vitamin D level was 17.9 ng/mL, indicating widespread deficiency. There was no significant difference in vitamin D levels between patients with autoimmune and non-autoimmune hypothyroidism ( *p* = 0.860), suggesting that vitamin D levels are independent of autoimmune hypothyroidism status. Additionally, there was no significant relationship between vitamin D levels and TSH ( *ρ* = −0.119, *p* = 0.148) or FT4 (ρ = 0.128, *p* = 0.123). Age showed a modest negative correlation with TSH levels ( *ρ* = −0.067, *p* = 0.416) and a positive but nonsignificant correlation with FT4 levels ( *ρ* = 0.024, *p* = 0.775).

**Conclusion:**

Serum vitamin D levels do not significantly differ between patients with autoimmune and non-autoimmune hypothyroidism, nor do they correlate with TSH levels. Further studies are needed to evaluate vitamin D status in these patient groups.

## Introduction

Vitamin D is a steroid molecule synthesized mainly under the skin upon exposure to ultraviolet B (UVB) light. 1 Other sources include dietary intake and supplementation. 2 Once synthesized or ingested, the liver converts it to 25-hydroxyvitamin D (25(OH)D _2_ or 25(OH)D _3_ ) through the process of hydroxylation, then renal proximal convoluted tubule cells convert it to the bioactive hormone (1,25(OH) _2_ D or calcitriol) by 1α-hydroxylase. 3

The human body's vitamin D status is best reflected by serum 25(OH)D _2_ because 1,25(OH)2D concentration could be normal or increased in some conditions, as in secondary hyperparathyroidism. 4 Vitamin D receptors are distributed across various body organs, including the thyroid gland, enabling it to exert diverse physiological functions. One of its primary roles is to regulate calcium and phosphorus homeostasis and support bone metabolism. 5 Studies have indicated that vitamin D is associated with nonskeletal diseases, such as metabolic syndrome, autoimmune diseases, cardiovascular diseases, and cancers. 6 7 Moreover, 1,25(OH) _2_ D has been demonstrated to regulate innate and adaptive immune systems. 8

In Jordan, vitamin D deficiency is highly prevalent and influenced by factors such as age, gender, and limited UVB exposure. 9 Decreased serum vitamin D is significantly linked with increased incidence of chronic autoimmune diseases, such as diabetes mellitus type 1 (DM 1), celiac disease, inflammatory bowel diseases, systemic lupus erythematosus, and multiple sclerosis. 10 11

Hypothyroidism is a condition in which the thyroid gland is underactive, resulting in a deficiency of thyroid hormones triiodothyronine (T3) and thyroxine (T4). 12 Etiology of hypothyroidism may be congenital or acquired. 13 In our research, we focus mainly on acquired causes, which can be classified as either autoimmune (e.g., Hashimoto's thyroiditis or less commonly atrophic thyroiditis) or non-autoimmune, which include iodine deficiency, transient thyroiditis (e.g., postviral or bacterial infection), and iatrogenic causes. 14

Hashimoto's thyroiditis, the most common cause of autoimmune hypothyroidism, is characterized by elevated thyroid peroxidase (TPO) and thyroglobulin (TG) antibodies. 15 The etiology of Hashimoto's thyroiditis is multifactorial, involving genetic predisposition, environmental factors, and immune dysregulation. 16

The relationship between vitamin D status and autoimmune hypothyroidism is still controversial. While some studies have demonstrated a significant association between low vitamin D levels and high antibody titers in Hashimoto's thyroiditis, others reported no such correlation. 17 18 19

To date, no comprehensive study has been performed in Jordan to compare serum vitamin D levels among patients with autoimmune (mainly Hashimoto's thyroiditis) and non-autoimmune hypothyroidism, which is defined as an elevation in the thyroid-stimulating hormone (TSH) levels with the absence of thyroid antibodies. This gap in the literature needs to be addressed to provide more information on this topic and to provide a better understanding of the medical field.

Our study aims to address this discrepancy by comparing serum vitamin D levels (25(OH)D _2_ ) between patients with autoimmune hypothyroidism and those with non-autoimmune hypothyroidism and evaluating the correlation between vitamin D levels and thyroid function (TSH and free T4 levels) in both groups.

## Methods

### Study Design and Ethical Approval

This retrospective observational study utilized medical records from the Jordanian Royal Medical Services (JRMS) in the period from January 2023 to November 2024. The study received approval from the Institutional Review Board at the JRMS.

### Study Population

The study population included patients diagnosed with hypothyroidism during the specified period. Of the initial 240 participants screened in the study, 90 were excluded due to exclusion criteria, yielding a final sample of 150.

Participants were categorized into two groups based on their thyroid antibody profiles. The first group, autoimmune hypothyroidism, was diagnosed based on elevated thyroid antibodies, specifically anti-TPO or anti-TG. The second group, non-autoimmune hypothyroidism, was defined by elevated TSH levels with the absence of thyroid antibodies.

### Inclusion and Exclusion Criteria

Inclusion criteria required patients to have documented hypothyroidism, defined as elevated TSH levels with low or normal free T4 (FT4) levels, a recent serum 25(OH)D _2_ measurement, and stable doses of levothyroxine or other thyroid medications for at least 6 months.

Patients were excluded from the study if they had other autoimmune diseases, chronic kidney disease, central hypothyroidism, liver disease, pregnancy, or malabsorption syndromes. Additionally, patients were excluded if they had recently used vitamin D supplements, chronic medication use interfering with vitamin D metabolism (e.g., anticonvulsants), parathyroid gland abnormalities, granulomatous diseases, DM, immunodeficiency, malignancy, chronic infections, chronic diseases, or metabolic bone diseases.

### Laboratory Measurements

All patients included in the study underwent standardized laboratory tests conducted at the central JRMS laboratory, which included the following parameters: serum TSH, FT4, anti-TPO, and anti-TG antibodies were measured using a chemiluminescence immunoassay. Serum 25(OH)D _2_ levels were assessed using a fluorescent enzyme immunoassay, both performed in the same laboratory using the “Tosoh AIA-900” analyzer to ensure consistency.

### Outcome Ranges

The reference ranges used for the study were as follows: vitamin D status was categorized as normal (≥ 30 ng/mL), insufficient (20–29 ng/mL), or deficient (< 20 ng/mL). The thyroid function test was defined as normal with TSH levels ranging from 0.27 to 4.2 µIU/mL. And normal FT4 levels ranging from 0.93 to 1.7 ng/dL. Primary hypothyroidism was characterized by elevated TSH levels with low or normal FT4 levels. Anti-TPO and anti-TG antibody levels above 34 and 115 IU/mL, respectively, were considered positive, indicating a diagnosis of autoimmune hypothyroidism.

### Statistical Analysis

Statistical analysis was performed using Jamovi version 2.3.28. Descriptive statistics were conducted for both quantitative variables (age, vitamin D, TSH, T4) and categorical variables (gender, cause of hypothyroidism, TPO-antibody [Ab], TG-Ab). Chi-square tests were used to compare categorical variables based on gender. Point biserial correlations were used to analyze the relationships between quantitative variables (age and vitamin D) and categorical variables. Spearman's correlations were applied to assess relationships among the quantitative variables. Logistic regression analysis was performed for autoimmune hypothyroidism, while linear regression analysis was used for TSH levels. A *p* -value of less than 0.05 was considered statistically significant.

## Results

### Descriptive Statistics and Frequencies

A total of 150 participants were included in the study; their ages ranged from 5 to 76 years old, with a mean age of 39.2 years (standard deviation [SD] = 14.5) and a median age of 39.5 years. The range of vitamin D values was 3.00 to 69.2 ng/mL, with a median of 17.3 ng/mL and a mean of 17.9 ng/mL (SD = 10.6). The range of TSH levels was 1.52 to 160 µIU/mL, with a mean of 15.0 µIU/mL (SD = 23.6), and a median of 6.66 µIU/mL. The range of FT4 levels was 0.039 to 1.90 ng/dL, with a mean of 1.03 ng/dL (SD = 0.295) and a median of 1.04 ng/dL. Age ( *p* = 0.186) and T4 levels ( *p* = 0.003) were roughly normally distributed, according to Shapiro–Wilk tests, while vitamin D ( *p* < 0.001) and TSH ( *p* < 0.001) were not.

Out of the total participants, 82% were female ( *n* = 123). Autoimmune hypothyroidism was observed in 41.3% of participants ( *n* = 62), whereas 58.7% ( *n* = 88) had non-autoimmune hypothyroidism. TPO antibodies (TPO-Ab) were positive in 36.0% of participants ( *n* = 54), with 26.0% ( *n* = 39) being female and 10.0% ( *n* = 15) male. Similarly, TG-Ab were positive in 20.0% of participants ( *n* = 30), comprising 14.7% ( *n* = 22) females and 5.3% ( *n* = 8) males. No significant associations were found between gender and autoantibody positivity, as shown in Table 1 .

**Table 1:** Baseline characteristics of participants

Variable	
**Age (y)**	
** Mean** ** Median** ** SD** ** Range**	39.2 39.5 14.5 5–76
**Vitamin D (ng/mL)**	
** Mean** ** Median** ** SD** ** Range**	17.9 17.3 10.6 3–69.2
**TSH (µIU/mL)**	
** Mean** ** Median** ** SD** ** Range**	15.0 6.66 23.6 1.52–160
**FT4 (ng/dL)**	
** Mean** ** Median** ** SD** ** Range**	1.03 1.04 0.295 0.0390–1.9
**Gender**	
** Male** ** Female**	27 (18%) 123 (82%)
**Cause of** hypothyroidism	
** Autoimmune** ** Non-autoimmune**	62 (41.3%) 88 (58.7%)
**Thyroid peroxidase antibodies,** ***n*** ** = 150**	
** Positive** ** Negative**	54 (36.0%) 96 (64.0%)
**Thyroglobulin antibodies,** ***n*** ** = 150**	
** Positive** ** Negative**	30 (20.0%) 120 (80.0%)

Abbreviations: FT4, free thyroxine; SD, standard deviation; TSH, thyroid-stimulating hormone.

### Gender, Autoimmune Hypothyroidism, and Vitamin D Levels

Autoimmune hypothyroidism was identified in 16 males (59.3% of males) and 46 females (37.4% of females). A chi-square test revealed a statistically significant association between gender and autoimmune hypothyroidism (chi-square = 4.36, *p* = 0.037), suggesting a higher prevalence among males in this cohort. Vitamin D levels did not differ significantly between those with and without autoimmune hypothyroidism, as determined by a Mann–Whitney *U* test ( *U* = 2636, *p* = 0.725), indicating that vitamin D levels are not associated with autoimmune hypothyroidism status ( Table 2 ).

**Table 2:** Associations of age, vitamin D, gender, and antibodies

Analysis	Test statistic	*p* -Value
**Autoimmune** hypothyroidism		
**- Age**	*t* = 1.53	0.128
**- Vitamin D**	*U* = 2636	0.725
**TPO** antibodies		
**- Age**	*t* = −0.160	0.111
**- Vitamin D**	*U* = 2456	0.594
**T** G antibodies		
**- Age**	*t* = −2.43	0.016
**- Vitamin D**	*U* = 1702	0.645
**Gender-** based comparisons		
**Autoimmune** hypothyroidism		
**- Male: Yes (16), No (11)**		
**- Female: Yes (46), No (77)**	χ ^2^ = 4.36	0.037
**TPO-Ab**		
**- Male:** positive **(15),** negative **(12)**		
**- Female:** positive **(39),** negative **(84)**	χ ^2^ = 5.47	0.019
**T** G **-Ab**		
**- Male:** positive **(8),** negative **(19)**		
**- Female:** positive **(22),** negative **(101)**	χ ^2^ = 1.91	0.167
**Spearman** correlations		
**- Age vs** . vitamin **D**	*r* = 0.215	0.008
**- Age vs** . **TSH**	*r* = −0.067	0.416
**- Age vs** . **FT4**	*r* = 0.024	0.775
**- Vitamin D vs** . **TSH**	*r* = −0.108	0.189
**- Vitamin D vs** . **FT4**	*r* = 0.115	0.164
**- TSH vs** . **FT4**	*r* = −0.343	< 0.001

Abbreviations: Ab, antibody; FT4, free thyroxine; TG, thyroglobulin; TPO, thyroid peroxidase; TSH, thyroid-stimulating hormone.

### Autoantibodies and Demographic Associations

TPO antibody positivity was more common in females (39 positive, 84 negative) compared with males (15 positive, 12 negative), and this difference was statistically significant (chi-square = 5.47, *p* = 0.019). In contrast, TG-Ab positivity did not significantly differ by gender (chi-square = 1.91, *p* = 0.167). When analyzing associations with age, a significant difference was found for TG-Ab, with younger individuals more likely to test positive ( *t* = −2.43, *p* = 0.016). No significant age differences were found for TPO antibodies ( *t* = −0.16, *p* = 0.111) or autoimmune hypothyroidism ( *t* = 1.53, *p* = 0.128).

### Correlations

Age and vitamin D levels were weakly and positively correlated ( *ρ* = 0.215, *p* = 0.008). No significant correlations were found between vitamin D and TSH ( *ρ* = −0.108, *p* = 0.189), or between vitamin D and FT4 ( *ρ* = 0.115, *p* = 0.164). TSH and FT4 were moderately and negatively correlated ( *ρ* = −0.343, *p* < 0.001). Other correlations, including those between age and thyroid function markers, were not statistically significant ( Table 2 ).

### Logistic and Linear Regression

**Fig. 1:**
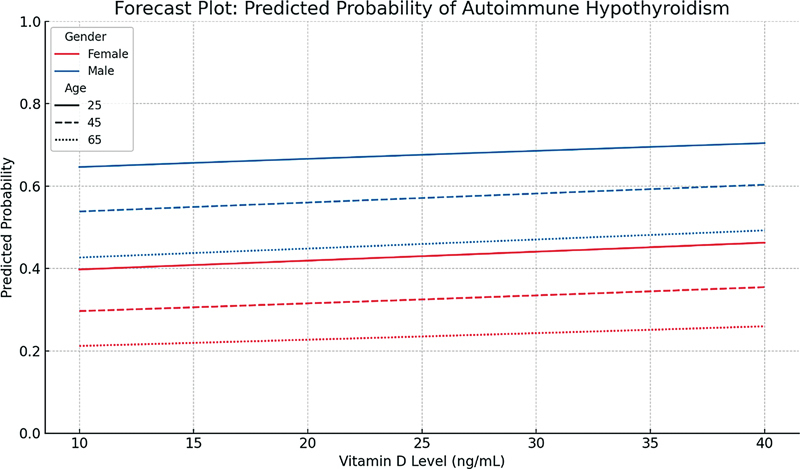
Logistic regression analysis of predictors for autoimmune hypothyroidism.

Fig. 1 shows a forecast plot of predictors for autoimmune hypothyroidism, focusing on how the probability of developing the condition changes with varying vitamin D levels, stratified by gender and age. In this plot, the predicted probabilities are modeled based on logistic regression estimates, with TSH and FT4 levels held constant. The plot compares males and females across three age groups—25, 45, and 65 years—allowing for a clearer visualization of age and gender effects. The curves show that males generally have a higher predicted probability of autoimmune hypothyroidism than females at similar levels of vitamin D, reflecting the significant association found in the regression analysis ( *p* = 0.028). As age increases, the predicted probability slightly decreases, consistent with the negative estimate for age, though it did not reach conventional significance ( *p* = 0.090). Vitamin D levels, despite being included as a continuous predictor, exhibit minimal effect on the outcome, which aligns with its nonsignificant role in the regression model ( *p* = 0.602). Overall, the plot helps illustrate the modeled relationships between key predictors and autoimmune hypothyroidism, offering a visual summary of the interplay between gender, age, and vitamin D in shaping risk estimates. Linear regression modeling explained 8.5% of the variance in vitamin D levels ( *R* ^2^ = 0.0850). Age was the only significant predictor ( *β* = 0.1617, 95% confidence interval [0.0372–0.286], *p* = 0.011), while TSH, FT4, TPO-Ab status, TG-Ab status, and gender were not significantly associated with vitamin D ( Tables 3 and 4 ).

**Table 3:** Logistic regression analysis for autoimmune hypothyroidism

Predictor	Estimate	Odds ratio	95% CI (lower–upper)	*p* -Value
Logistic regression for autoimmune hypothyroidism				
- Intercept	1.60966	5.001	−0.4719 to 3.6912	0.130
- Vitamin D (ng/mL)	0.00884	1.009	−0.0244 to 0.04205	0.602
- Age	−0.02246	0.978	−0.0484 to −0.00353	0.090
- TSH	−0.00669	0.993	−0.0251 to 0.01168	0.475
- FT4	−1.282276	0.277	−2.7667 to 0.2012	0.090
- Gender (male vs. Female)	1.01798	2.768	0.1101 to 1.9259	0.028

Abbreviations: CI, confidence interval; FT4, free thyroxine; TSH, thyroid-stimulating hormone.

**Table 4:** Linear regression analysis of vitamin D predictors

Predictor	Estimate	95% CI (lower–upper)	*p* -Value
**Intercept**	7.6763	−2.2176 to 17.570	0.127
**Age**	0.1617	0.0372 to 0.286	0.011
**TSH (µIU/mL)**	0.0283	−0.0620 to 0.119	0.536
F **T4 (ng/dL)**	3.5978	−3.5042 to 10.700	0.318
**TPO-Ab (Negative vs** . **Positive)**	−1.5188	−5.4211 to 2.384	0.443
**T** G **-Ab (Negative vs** . **Positive)**	0.8754	−3.8084 to 5.559	0.712

Abbreviations: Ab, antibody; CI, confidence interval; FT4, free thyroxine; TG, thyroglobulin; TPO, thyroid peroxidase; TSH, thyroid-stimulating hormone.

## Discussion

The objective of this study was to explore the potential association between serum vitamin D levels and autoimmune versus non-autoimmune hypothyroidism in the Jordanian population. The association between reduced serum vitamin D and autoimmune diseases is well-recognized in the literature. Bellastella et al documented that patients diagnosed with autoimmune disorders exhibited significantly lower levels of 25(OH)D compared with healthy controls, proposing a potential role of vitamin D deficiency in autoimmune pathogenesis. 20 Similarly, Muscogiuri et al emphasized that vitamin D deficiency is common in autoimmune thyroid diseases, including Hashimoto's thyroiditis, and highlighted its potential role in modulating immune responses. 21 Despite this fact, our findings did not demonstrate a significant association between vitamin D levels and the type of hypothyroidism; *p* -value for autoimmune hypothyroidism = 0.602. Findings of a study conducted by Goswami et al, in the Asian Indian population, established a weak association between vitamin D levels in autoimmune thyroiditis patients and healthy controls, suggesting variability in the degree of association depending on the study population and methodology. 22

We observed a nonsignificant correlation between vitamin D levels and TSH levels ( *p* = 0.536), indicating that lower vitamin D levels may not be associated with poorer thyroid function. Two studies conducted by Vieira et al and Bozkurt et al reported lower vitamin D levels in patients with Hashimoto's thyroiditis compared with healthy controls, with a correlation between the severity of vitamin D deficiency and disease markers such as elevated TSH and thyroid antibody titers in autoimmune thyroiditis. 23 24 In his study, Mirhosseini et al suggested that although vitamin D supplementation may improve thyroid antibody titers in autoimmune thyroid diseases, its impact on thyroid hormone levels and clinical outcomes remains uncertain, warranting further investigation. 25

The immunomodulatory properties of vitamin D have been widely studied, with evidence suggesting its role in regulating T cell responses and reducing inflammation, particularly in autoimmune diseases such as Hashimoto's thyroiditis. 26 27 Nonetheless, our findings suggest that vitamin D levels may not differentiate between autoimmune and non-autoimmune hypothyroidism within a clinical population. Also, while autoimmune hypothyroidism is reported to be more prevalent in males, compared with previous studies reported in the literature. 28 Several factors could account for this discrepancy. First, differences in study design, population demographics, and the most importantly is our small sample size, in addition to selection bias, which may influence results. While previous studies often compared autoimmune hypothyroidism patients to healthy individuals, our study compared two hypothyroid subgroups. This distinction eliminates the confounding effects of thyroid function itself but may dilute the observable impact of vitamin D on autoimmunity. 26 29 30

Second, the retrospective nature of our study and reliance on existing medical records may have introduced variability in the timing of vitamin D measurements, seasonal fluctuations, and confounding variables such as dietary habits, sun exposure, and comorbidities. Finally, the pathophysiological mechanisms underlying hypothyroidism may involve multifactorial processes that are not solely dependent on vitamin D levels. For example, genetic predispositions, environmental triggers, and other micronutrient deficiencies might play a larger role in determining autoimmune thyroid disease than previously assumed. 31

This study has certain limitations that need to be recognized. The retrospective design, the subgroup analysis of TPO-Ab and TG-Ab was limited by a smaller sample size of 150, which may reduce statistical power to detect associations, seasonal variation, or sun exposure, which could influence the results, and a lack of control over confounding variables may limit the generalizability of our findings. Additionally, the sample size may not capture subtle group differences. Prospective studies with standardized vitamin D assessments and consideration of genetic and lifestyle factors are warranted.

## Conclusion

Given the lack of significant association between vitamin D levels and autoimmune/non-autoimmune hypothyroidism or TSH values, future studies should prioritize longitudinal studies and more diverse populations to elucidate potential relationships. These efforts could help reconcile conflicting evidence and determine whether vitamin D supplementation plays any role in thyroid autoimmunity prevention or management. Additionally, exploring confounding factors (e.g., sun exposure, dietary habits) or threshold effects of vitamin D may provide further insights into the complexity of autoimmune thyroid disease.
